# Large family with both parents affected by distinct BRCA1 mutations: implications for genetic testing

**DOI:** 10.1186/1897-4287-7-2

**Published:** 2009-01-26

**Authors:** Anna P Sokolenko, Dmitry A Voskresenskiy, Aglaya G Iyevleva, Elena M Bit-Sava, Nadezhda I Gutkina, Maxim S Anisimenko, Nathalia Yu Sherina, Nathalia V Mitiushkina, Yulia M Ulibina, Olga S Yatsuk, Olga A Zaitseva, Evgeny N Suspitsin, Alexandr V Togo, Valery A Pospelov, Sergey P Kovalenko, Vladimir F Semiglazov, Evgeny N Imyanitov

**Affiliations:** 1 NN Petrov Institute of Oncology, St.-Petersburg, Russia; 2 Institute of Molecular Biology and Biophysics, Novosibirsk, Russia; 3 Institute of Cytology, St.-Petersburg, Russia

## Abstract

Although the probability of both parents being affected by BRCA1 mutations is not negligible, such families have not been systematically described in the literature. Here we present a large breast-ovarian cancer family, where 3 sisters and 1 half-sister inherited maternal BRCA1 5382insC mutation while the remaining 2 sisters carried paternal BRCA1 1629delC allele. No BRCA1 homozygous mutations has been detected, that is consistent with the data on lethality of BRCA1 knockout mice. This report exemplifies that the identification of a single cancer-predisposing mutation within the index patient may not be sufficient in some circumstances. Ideally, all family members affected by breast or ovarian tumor disease have to be subjected to the DNA testing, and failure to detect the mutation in any of them calls for the search of the second cancer-associated allele.

## Case presentation

BRCA1 and BRCA2 mutations occur in approximately 0.2–1% healthy subjects, 5% non-selected breast cancer (BC) patients, 15% consecutive ovarian cancer (OC) cases, and 25% females with clinical features of hereditary breast-ovarian cancer (HBOC) syndrome [1-8]. Given low populational frequency and high penetrance of deleterious BRCA1 and BRCA2 mutations, only one of the parent of the affected proband is usually suspected to contain this genetic lesion. Nevertheless, the probability of both parents being a carrier of BRCA1 or BRCA2 mutation is not negligible. If we consider random healthy couples, this estimate would be equal to 0.2–1% × 0.2–1% = 0.0004–0.01% (i.e., 1:250000 - 1:10000). This probability is significantly increased if we deal with already affected females. Indeed, the most conservative calculation would imply that the chance to detect a mutation in one of the parents of the patient is the same as in the patient herself, whereas the probability for the remaining parent to be affected by BRCA1 or BRCA2 defect is similar to the populational frequency of the latter. Therefore, approximately 0.01–0.05% (i.e., 1:10000 - 1:2000) of BC patients, 0.03 – 0.15% (i.e., 1:3333-1:667) of OC patients, and 0.05–0.25% (i.e., 1:2000 - 1:400) of women with HOBC have both parents carrying BRCA1 or BRCA2 mutation. Coincident occurrence of BRCA1 heterozygosity in mother and father is particularly likely if both parents of the patient report a strong family history of the breast-ovarian cancer disease, however these situations are exceptionally rare due to gender-specific penetrance of BRCA1 and BRCA2 alterations and lack of explicit information in the most of pedigrees.

**Figure 1:**
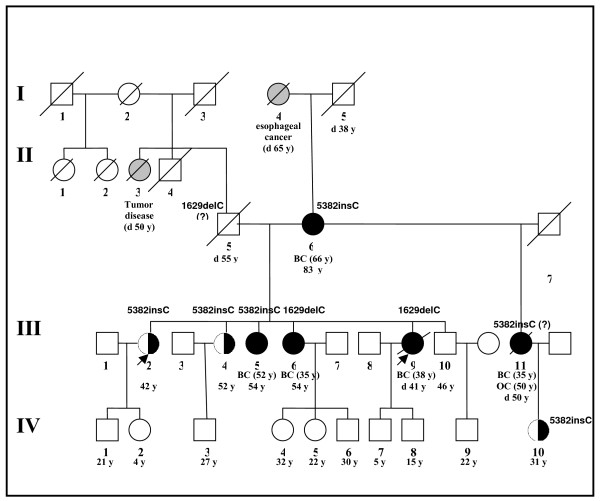
**Pedigree of the breast-ovarian cancer family with 2 BRCA1 mutations**. Circles and squares indicate females and males, respectively. Crossed symbols are used for deceased subjects; age at death is given after the letter "d", otherwise current age is provided. Black circles correspond to females affected by breast or ovarian cancer; half-filled circles are used for yet healthy BRCA1 mutation carriers; gray symbols depict subjects with unknown or non-breast-ovarian tumors. BC – breast cancer; OC – ovarian cancer; age at cancer diagnosis is given in brackets. All tested subjects carried either BRCA1 5382insC or BRCA1 1629delC allele, i.e. missing information on the BRCA1 status indicates that DNA from this family member has not been analyzed. Index cases are designated by arrows (see the text).

Here we report an unique family with multiple affected members and 2 distinct BRCA1 mutations identified. Breast cancer patient III.9 was forwarded to a genetic counselor in the year 2002 because of pronounced family history (Fig. 1). She underwent DNA testing for Russian founder mutations [8], however no genetic lesion has been identified. DNA sequencing was not accessible to us at that time, however, given an unusually strong family history of breast-ovarian cancer and large number of yet healthy female relatives, we desperately attempted to find a facility who would agree to perform a full-length BRCA1 and BRCA2 analysis for this particular patient. Unfortunately, this effort failed to succeed. The patient and her only sister who accepted the invitation for genetic counseling (III.5, healthy by then) were informed that their family is likely to suffer from hereditary cancer syndrome, and the failure to identify a causative mutation may be related to current technical limitations. Therefore, we recommended tight diagnostic monitoring for BC and OC, as well as consideration of comprehensive analysis of breast cancer genes as soon as it becomes available. In the year 2007, healthy female III.2 was forwarded to DNA testing by her mammologist because of cancer family history concerns. "Founder" test-panel [8] revealed the presence of BRCA1 5382insC mutation, therefore she was advised to consult with a cancer geneticist. Conversation with this women revealed that she belonged to the same family as the patient III.9, however she was unaware about genetic counseling applied to her sisters III.9 and III.5. Then, BRCA1 5382insC mutation testing was performed for her affected mother (II.6, BC at age 66), her sisters (III.4, healthy; III.5, affected by BC at age 52, i.e. after the initial counseling in the year 2002; III.6, BC at age 35), and her healthy 31-years-old nephew whose mother (III.11) survived BC at age 35 but died from OC at 50. All these relatives were found to carry BRCA1 5382insC mutation, with the exception of BC patient III.6. Since III.6 was already a second family member, who acquired cancer disease but was negative for founder mutation test, sequencing analysis was applied to DNA samples III.6 and III.9; it identified second BRCA1 mutation, 1629delC.

Since 2 sisters from this family carry BRCA1 1629delC, it is highly unlikely that these mutations appeared de novo; instead, it is nearly certain that the father of these sisters (II.5) was a carrier of this allele. We could not validate this assumption, because this man died a long time ago. Similarly, patient III.11 was likely to carry BRCA1 5382insC mutation, but this could not be confirmed.

Homozygosity for BRCA1 inactivation was reported previously in a single human [9], although this observation was later suggested to be a technical artifact [10]. We conducted testing for both BRCA1 5382insC and 1629delC alleles in all available DNA samples, and failed to detect any biallelic defect. Taken together with the only available publication on BRCA1 mutation occurring in both parents [11] and a large body of evidence for early embryonic lethality of BRCA1 knockout mice [12-14], our data provide additional support to the common statement on the lack of viability of BRCA1-null homozygotes. This contrasts to the situation with another breast-ovarian cancer gene, BRCA2, whose homozygous inactivation leads to Fanconi anemia but is not absolutely lethal [15].

## Conclusion

While families harboring both BRCA1 and BRCA2 mutations have been repeatedly presented in the literature [16], there is no systematic analysis of the pedigrees with both parents carrying distinct alterations in the BRCA1 gene [11]. Our observation calls for the same caution as was formulated in a series of articles describing double mutations for BRCA1 and BRCA2 [16]. First, in the communities with strong founder effect, relaxed criteria for subjects selection should be used when considering the administration of appropriate non-expensive PCR tests; it is highly recommended that all BC and OC patients, and perhaps even healthy middle-aged females, have to be offered the opportunity of the DNA analysis [3,6,8,16]. Secondly, the possibility of existence of disease phenocopies within the same family has to be always remembered; therefore, failure to identify breast-ovarian cancer gene mutation in the index case does not exclude the possibility of identification of BRCA defect in other affected members of a given pedigree. Ideally, all affected members of the family have to undergo DNA testing at least for the presence of founder mutations [16].

## Abbreviations

BC: breast cancer; OC: ovarian cancer; HBOC: hereditary breast-ovarian cancer (HBOC) syndrome 

## Consent

Written informed consent was obtained from the alive memebrs of the described family for the publication of this case report. Copies of the written consent are available for review by the Editor-in-Chief of this journal.

## Competing interests

The authors declare that they have no competing interests.

## Authors' contributions

VFS and ENI were responsible for the conception of the study. APS, DAV, AGI, EMBS, NIU, MSA, NYS, NVM, YMU, OSY, OAZ, ENS, AVT, VAP, SPK were responsible for data collection. APS, DAV, SPK were responsible for data analysis. ENI wrote the manuscript. All authors approved the final version.
